# Epoxy-Anhydride Vitrimers from Aminoglycidyl Resins with High Glass Transition Temperature and Efficient Stress Relaxation

**DOI:** 10.3390/polym12051148

**Published:** 2020-05-17

**Authors:** Michael Giebler, Clemens Sperling, Simon Kaiser, Ivica Duretek, Sandra Schlögl

**Affiliations:** 1 Polymer Competence Center Leoben GmbH, Roseggerstrasse 12, A-8700 Leoben, Austria; michael.giebler@pccl.at (M.G.); clemens.sperling@stud.unileoben.ac.at (C.S.); simon.kaiser@pccl.at (S.K.); 2 Chair of Polymer Processing, Montanuniversitaet Leoben, Otto Gloeckel-Strasse 2, A-8700 Leoben, Austria; ivica.duretek@unileoben.ac.at

**Keywords:** vitrimers, glass transition temperature, aminoglycidyl resins

## Abstract

Epoxy-anhydride vitrimers are covalent adaptable networks, which undergo associative bond exchange reactions at elevated temperature. Their service temperature is influenced by the glass transition temperature (*T*_g_) as well as the topology freezing transition temperature (*T*_v_), at which the covalent bond exchange reactions become significantly fast. The present work highlights the design of high-*T*_g_ epoxy-anhydride vitrimers that comprise an efficient stress relaxation at elevated temperature. Networks are prepared by thermally curing aminoglycidyl monomers with glutaric anhydride in different stoichiometric ratios. The tertiary amine groups present in the structure of the aminoglycidyl derivatives not only accelerate the curing reaction but also catalyse the transesterification reaction above *T*_v_, as shown in stress relaxation measurements. The topology rearrangements render the networks recyclable, which is demonstrated by reprocessing a grinded powder of the cured materials in a hot press. The epoxy-anhydride vitrimers are characterised by a high *T*_g_ (up to 140 °C) and an adequate storage modulus at 25 °C (~2.5 GPa), which makes them interesting candidates for structural applications operating at high service temperature.

## 1. Introduction

Epoxy resins have a wide range of industrial applications but suffer from a lack of recyclability due to their permanently cross-linked network structure. This changed in 2011 due to the pioneering work of Leibler and his group, who introduced epoxy-based vitrimers, which represent a new class of covalent adaptable networks (CANs) [1]. Vitrimers are thermosets, which are capable of changing their network connectivity by thermally activated bond exchange reactions [2]. The exchange rate of those reactions increases with temperature and becomes macroscopically relevant above the topology freezing transition temperature (*T*_v_). Below *T*_v_, the network structure is frozen, whilst above *T*_v_, the network is able to flow macroscopically and fully relax stresses, behaving like a viscoelastic liquid [3,4,5,6]. This enables the introduction of new material properties in epoxy-based networks, such as self-healing and recyclability [7,8,9,10].

For vitrimers relying on transesterification reactions, an appropriate catalyst is often required to accelerate the thermally activated bond exchange reactions [1]. Previous studies revealed that catalyst type and concentration influence the *T*_v_, and several types have been introduced [4]. The most popular types are organic zinc salts, such as zinc acetylacetonate and zinc acetate [1,11,12]. The organic guanidine base 1,5,7-triazabicyclo[4.4.0]dec-5-ene is another effective transesterification catalyst, which has been often used in the design of epoxy-based vitrimeric systems [13,14]. Recently, Williams and co-workers reported that covalently bonded tertiary amines have similar capabilities in catalysing transesterification reactions as the catalysts commonly applied in vitrimers [4]. They reacted primary and secondary amines with an excess of epoxy monomer followed by a curing of the pre-reacted epoxy monomer with a stoichiometric amount of carboxylic acid groups. Dynamic networks with properties comparable to classically catalysed vitrimers were obtained.

Along with the type and concentration of the catalyst, the exchange reaction kinetics in vitrimers is governed by the number of hydroxyl and ester groups in the networks [15,16]. Epoxy-anhydride systems cured with an equimolar ratio of epoxide to anhydride groups are not susceptible to transesterification reactions due the absence of hydroxyl groups [17]. Interestingly, a high number of ester moieties enables exchange reactions even in the absence of ample hydroxyl groups [18]. In addition, Hayashi et al. showed that stress relaxation increases with rising crosslink density in polyester vitrimers, whilst the number of free hydroxyl groups was kept constant [15].

To date, a large variety of different vitrimer systems has been published and attempts have been made to transfer these new materials to industrial applications [19]. However, to the best of our knowledge, the majority of the reported vitrimer systems are elastomeric networks at room temperature, having glass transition temperatures (*T*_g_) below 100 °C, which limits their applicability in structural components [20,21,22,23,24]. In contrast, high-T_g_ vitrimer systems often suffer from slow and insufficient transesterification reactions [1,4,25]. This is explained by the lack of free hydroxyl moieties and the low network mobility. In particular, epoxy-anhydride and epoxy-acid vitrimers are typically prepared from non-stoichiometric ratios between the chemical functions of monomer and hardener. A lower content of either acyl groups or carboxylic acid moieties with respect to the hydroxyl groups of the epoxy monomers is employed to ensure a certain number of free hydroxyl moieties necessary for fast transesterifications [17,23]. The resulting networks are characterized by a lower cross-link density, which compromises the *T*_g_. Studies on vitrimers from bisphenol A diglycidyl ether and glutaric anhydride revealed that the *T*_g_ decreases from 68 to 59 °C by changing the stoichiometry of the resin formulation [17]. In addition, vitrimer networks are often built from epoxy monomers or hardeners with soft and flexible structures to ensure a high mobility of the network. Although soft systems perform well in terms of healing, welding and repairing, they suffer from a low *T*_g_.

An elegant approach towards high-*T*_g_ networks from bio-based resources was pursued by Zhang and co-workers, who synthesised a tri-functional aromatic epoxy monomer and cured it with methylhexahydrophthalic anhydride (MHHPA) in the presence of Zn(acac)_2_−H_2_O as catalyst [23]. The networks were characterised by a *T*_g_ of up to 187 °C and showed reasonable repair properties at elevated temperature (220 °C). They explained the good repair properties of these high-*T*_g_ networks by a sufficiently high number of hydroxyl groups, a relatively low rubbery modulus and the non-coplanar structure of the epoxy monomer, which facilitates the increase in mobility of the polymer chains with temperature.

**Figure 1:**
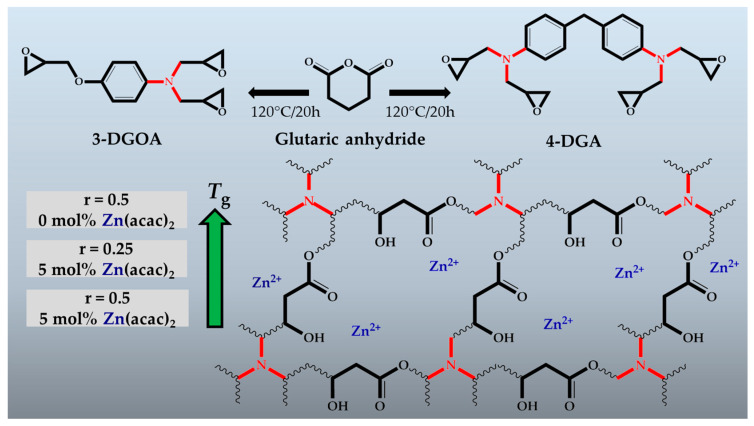
Epoxy-anhydride vitrimers obtained from 4,4′-methylenebis(*N*,*N*-diglycidylaniline) (4-DGA) and *N*,*N*-diglycidyl-4-glycidyloxyaniline (3-DGOA) with varying glass transition temperature (*T*_g_) prepared in the present study. *r* denotes the stoichiometric ratio, which was defined as *r* = anhydride equiv/epoxy equiv.

Inspired by this work, we transferred this concept to technically relevant epoxy monomers, which are well known for their ability to produce networks with high *T*_g_ (>200 °C) [26,27]. In particular, we prepared networks from the tetra-functional 4,4′-methylenebis(*N*,*N*-diglycidylaniline) (4-DGA) and the tri-functional *N*,*N*-diglycidyl-4-glycidyloxyaniline (3-DGOA) using glutaric anhydride as hardener (Figure 1). Compared to MHHPA, which is a typical hardener for high-performance epoxy-based composites, glutaric anhydride does not contain any rigid aromatic structures contributing to a high *T*_g_. However, MHHPA is listed as a substance of very high concern (SVHC) by the European Chemicals Agency, and considering future industrial applications, we chose glutaric anhydride as an alternative hardener. 4-DGA and 3-DGOA not only provide rigid benzene rings for tuning the *T*_g_ of the network, but also tertiary amine moieties, which are capable of catalysing both the curing and transesterification reaction of vitrimer systems. Without the addition of a conventional catalyst, networks with a *T*_g_ of 140 °C and a *T*_v_ of 200 °C were obtained, which showed remarkable stress relaxation, enabling the reprocessing and recycling of the materials.

## 2. Materials and Methods

### 2.1. Materials

*N*,*N*-diglycidyl-4-glycidyloxyaniline, 4,4′-methylenbis(*N*,*N*-diglycidylaniline), zinc(II)- acetylacetonate hydrate and glutaric anhydride (95%) were purchased from Sigma Aldrich (St. Lois, MO, USA). The chemicals were used as received. For preparative work, hazardous chemicals and solvents were employed. Reactions must be carried out in a fume hood, and protective clothes and goggles must be used.

### 2.2. Methods

#### 2.2.1. Preparation of Epoxy-Anhydride Vitrimers

For the preparation of the catalysed vitrimers, either 4,4′-methylenebis(*N*,*N*-diglycidylaniline) (4-DGA) or *N*,*N*-diglycidyl-4-glycidyloxyaniline (3-DGOA) were mixed with Zn(acac)_2_-H_2_O (at a concentration of 5 mol % related to the epoxy groups) in a PTFE beaker and heated to 100 °C. The formulation was stirred at 100 °C for 10 min to dissolve the catalyst. Glutaric anhydride was added stepwise and the formulation was stirred for another 10 min at 100 °C. Air bubbles and low-molecular substances (e.g., acetylacetone from the catalyst) were removed under vacuum for 10 min. For the non-catalysed systems, the formulation was prepared without the addition of Zn(acac)_2_-H_2_O. Table 1 summarises the composition of the systems under investigation.

#### 2.2.2. Fourier-Transform Infrared Spectroscopy (FT-IR)

In order to monitor the progress of the thermally induced ring-opening reaction between the epoxide and the anhydride hardener, FT-IR spectroscopy with a Vertex 70 spectrometer (Bruker, Billerica, MA, USA) was carried out. Sixteen scans were accumulated in transmission mode with a resolution of 4 cm^−1^. The areas of the IR absorption peaks were calculated with OPUS software (Version 7.0, Bruker, Billerica, MA, USA). For sample preparation, thin films were spin-cast from chloroform solutions (1 mg/mL) of the resin formulations (Table 1) on a Si wafer.

#### 2.2.3. Dynamic-Mechanical Thermal Analysis (DMTA)

For sample preparation, the resin formulations were poured into a pre-heated (120 °C) metal mold and cured for 20 h at 120 °C. Cured sheets with a thickness of 1.5 mm were obtained, from which rectangular test specimens (3 × 12 mm) were cut. DMTA experiments were carried out with a Mettler Toledo SDTA861e dynamic mechanical analyzer (Columbus, OH, USA) in tensile mode. A clamping length of 9 mm was chosen, and the measurements were performed in displacement-controlled oscillation of 2.5 µm amplitude at 1 Hz. The heating rate was 3 K/min from −50 to 230 °C.

#### 2.2.4. Thermogravimetric Analysis (TGA)

TGA experiments were performed on a Mettler Toledo (Columbus, OH, USA) TGA/DSC1 thermogravimetric analyzer. Measurements were done under nitrogen atmosphere. The samples were heated from 25 to 900 °C at a heating rate of 10 °C/min and the data were analysed with the STAR software of Mettler-Toledo (Columbus, OH, USA).

#### 2.2.5. Stress Relaxation

Stress relaxation was determined in torsion mode using an MCR 501 rheometer (Anton Paar, Graz, Austria) equipped with a rectangular torsion fixture. The heating chamber was continuously purged with nitrogen at a volume flow of 1 m³/h. Rectangular samples with a uniform thickness of 1.5 mm, a length of 30 mm and a width of 5 mm were heated to the desired measurement temperature (160–280 °C) and equilibrated for 20 min. A constant elongational force of 0.1 N was applied to ensure a stable position of the sample. After temperature equilibration, a deflection of 1% was applied and kept constant during the measurements, whilst the torque was monitored over time.

#### 2.2.6. Recycling of Vitrimers

The cured 3-DGOA-0.5-Zn and 4-DGA-0.5-Zn networks were grinded with a ball mill for 90 s and 30 Hz. The resulting powder was molded in discs with a diameter of 25 mm with a vacuum press (Collin, Ebersberg, Germany) applying a pressure of 5 bar for 40 min at 220 °C. In addition, molded test bars were prepared in a PVT 100 (SWO Polymertechnik, Krefeld, Germany) operating at 500 bar. The pressure was applied at 250 °C for 5 min.

## 3. Results and Discussion

### 3.1. Design and Curing of High-T_g_ Epoxy-Anhydride Vitrimers

Epoxy-anhydride networks were prepared by reacting either *N*,*N*-diglycidyl-4-glycidyloxyaniline (3-DGOA) or 4,4′-methylenbis(*N*,*N*-diglycidylaniline) (4-DGA) with glutaric anhydride at different stoichiometric ratios and catalyst contents (Table 1). The stoichiometric ratio *r* was defined as *r* = anhydride equiv/epoxy equiv and amounted to either 0.5 or 0.25. In technical applications, *r* typically ranges between 0.5 and 0.9 to obtain optimum mechanical and thermal properties of the thermosets [28]. However, aiming at the fabrication of hydroxyl ester networks with a high number of free –OH groups, we also prepared epoxy-anhydride systems with a high excess of epoxy groups (*r* = 0.25).

For the preparation of the Zn(acac)_2_−H_2_O catalysed epoxy-anhydride networks, the transesterification catalyst was dissolved in the particular aminoglycidyl monomer, which was preheated at 100 °C. Next, the anhydride was added stepwise, and after obtaining a homogeneous mixture the curing was carried out at 120 °C.

**Figure 2:**
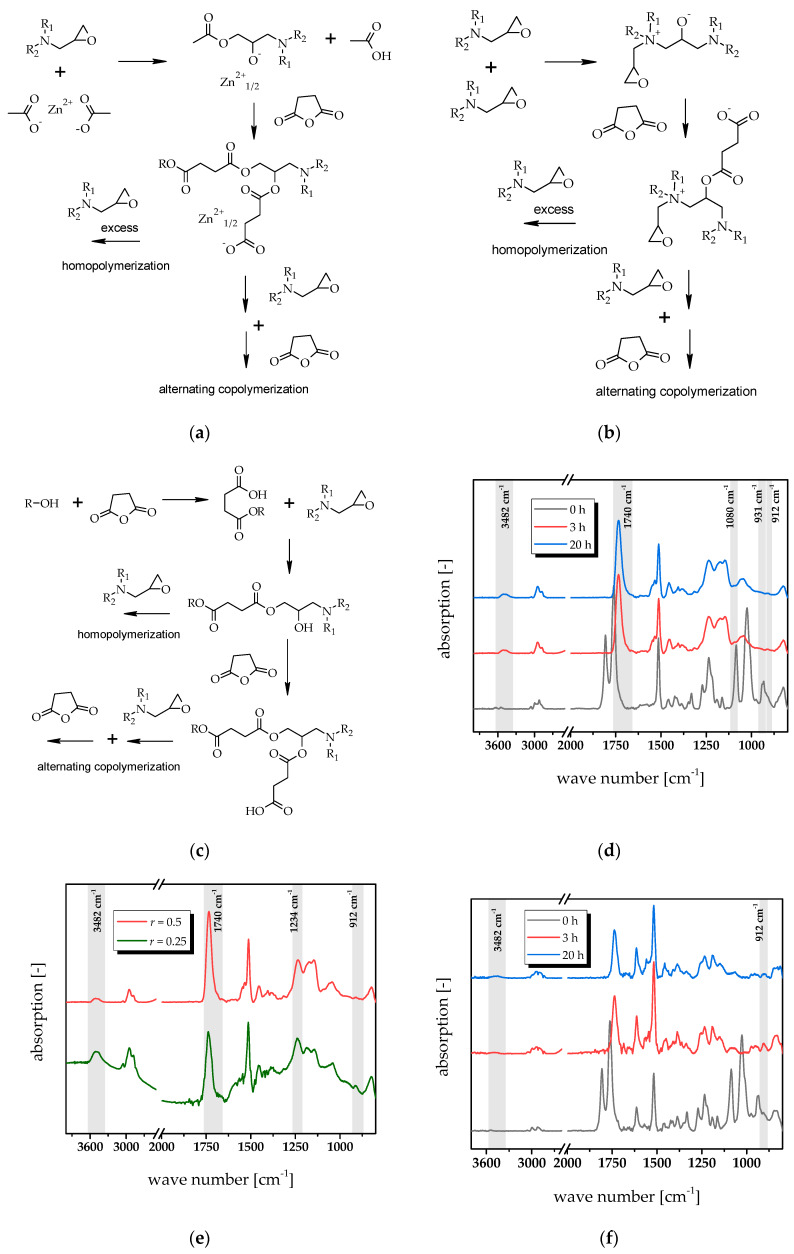
Mechanism of the ring opening reaction of epoxies with anhydrides in the presence of (**a**) zinc salts and (**b**) tertiary amines as catalysts or (**c**) without catalyst. (**d**) FT-IR spectra of catalysed 3-DGOA/glutaric anhydride systems cured with *r* = 0.5, prior to and after curing at 120 °C for 3 and 20 h. (**e**) FT-IR spectra of catalysed 3-DGOA/glutaric anhydride systems as a function of the stoichiometric ratio, after curing at 120 °C for 20 h. (**f**) FT-IR spectra of catalysed 4-DGA/glutaric anhydride systems cured with *r* = 0.5, prior to and after curing at 120 °C for 3 and 20 h.

The curing kinetics was characterised using FT-IR spectroscopy. Figure 2d shows the FT-IR spectra of 3-DGOA-0.5-Zn prepared with *r* = 0.5, prior to and after thermal curing at 120 °C. After a cure time of 3 h, the two bands (1860 and 1778 cm^−1^) related to carbonyl of the anhydride groups disappeared whilst a new band was observed at 1740 cm^−1^, which is attributed to the carbonyl group of the newly formed ester linkages. A broad absorption band appeared at 3482 cm^−1^, which is associated with the formation of hydroxyl groups. Moreover, the epoxy band at 910 cm^−1^ and the partly overlapping C–O absorption band (931 cm^−1^) of the anhydride hardener disappeared. Thus, the results demonstrate the successful formation of hydroxyl ester linkages via the nucleophilic ring-opening reaction between the aminoglycidyl epoxy monomers and glutaric anhydride. Full conversion of the functional groups is obtained in under 3 h, and no significant change in the bands was observed for extended curing of 20 h.

The fast curing can be explained by the catalytic effect of Zn(acac)_2_−H_2_O. It is well known that organic zinc salts take part in the curing reaction due to their nucleophilic nature [29,30]. By dissolving the zinc salt in the epoxy monomers, the Zn^2+^ ions are able to substitute the organic ligands with the oxygen of the epoxide ring. Subsequently, ring opening of the epoxide groups takes place, and after adding the anhydride, carboxylate-ions are formed (Figure 2a), which undergo a nucleophilic ring-opening reaction with the epoxy moieties. Ester groups and additional carboxylate anions are formed, which leads to a perpetuation of the ring-opening reaction. The reaction follows an anionic alternating copolymerization, yielding a polyester network. The ligands typically evaporate during solvation and curing and the Zn^2+^ ions remain in the cured network. In addition, water is released from the catalyst at high temperature, which further accelerates the reaction by hydrolysing the anhydride ring under the formation of carboxylic acids. Furthermore, it should be noted that a homopolymerisation of the epoxy groups can take place as an alternative reaction pathway at an excess of epoxy monomers and at low curing temperatures [27]. At 1080 cm^−1^, a small band is observed, which might be related to the C–O absorption band of ethers [31]. However, the competition between esterification and etherification cannot be clearly identified since C–O bands of the ester moieties at 1250 cm^−1^ overlap with ether bands (1200–1080 cm^−1^) in the fingerprint area.

Increasing the excess of epoxy moieties (catalysed formulations cured with *r* = 0.25), the IR absorption band of the –OH groups (3482 cm^−1^) significantly increases in cured samples, whilst the band area of the formed ester bonds (1740 cm^−1^) is less pronounced (Figure 2e). Full conversion of the anhydride moieties is observed, but the consumption of the epoxy groups is not complete, which is indicated by the high intensity of the C–O band at 1234 cm^−1^ and the remaining C–O band at 910 cm^−1^ [27]. The results clearly show the presence of non-reacted epoxy groups (~6%) giving rise to an incomplete homopolymerization of 3-DGOA. This is mainly due to diffusion limitations of the monomers above the gel point and which are typically observed in aromatic epoxies at high degrees of conversion (>90%) [32,33,34]. During the course of the curing reaction, the mobility of the monomers decreases due to the high viscosity of the matrix and the reaction becomes diffusion controlled since the diffusion of reactive species toward each other is hindered.

This behavior is even more pronounced in catalysed formulations with 4-DGA, since the epoxy monomers comprise a higher functionality and the rigid bi-functional aromatic structure makes them less mobile. Thus, even in 4-DGA networks cured with *r* = 0.5, a small amount of epoxy groups (~4%) remain unreacted after curing at 120 °C for 20 h (Figure 2f). In addition, the band area of the –OH groups (3482 cm^−1^) is smaller than in spectra of the cured 3-DGOA counterpart, indicating a lower number of –OH groups. Since a high number of OH groups is beneficial in the transesterification reaction, 4-DGA networks are expected to be less efficient in stress relaxation experiments [18].

In addition, non-catalysed networks from 3-DGOA and 4-DGA with *r* = 0.5 were prepared. In the absence of a catalyst, the curing of epoxy-anhydride systems follows a step growth reaction (Figure 2c) [32]. Traces of water or impurities with –OH groups start the reaction by opening the anhydride rings to form carboxylic acid groups. Once formed, the carboxylic acid groups undergo a nucleophilic ring opening reaction with the epoxy groups generating –OH groups, which react with further anhydride moieties [35]. For the curing of aminoglycidyl monomers, previous studies revealed that the tertiary amine groups of epoxy monomers can catalyse the reaction [27].

In the base catalysed ring-opening reaction, the tertiary amine groups open the epoxy rings under the formation of alkoxides (Figure 2b) [32,34,36]. The alkoxides react with anhydride groups yielding carboxylate anions, which are again able to open epoxy moieties. The reaction also follows an anionic alternating copolymerization and yields a polyester network. As with the zinc catalysed system, homopolymerisation of the epoxy moieties can occur in resin formulations with an excess of epoxy monomers [35].

From FT-IR data it can be obtained that the final monomer conversions of systems without Zn(acac)_2_−H_2_O are comparable to catalysed ones, albeit they are reached at longer curing times. This is in good agreement with previous work on epoxy-anhydride systems demonstrating that the catalyst mainly influences the cure rate but not the final monomer conversion [34]. To ensure maximum conversion of the monomers, which was monitored by the disappearance of the epoxy band at 910 cm^−1^, all networks were cured at 120 °C for 20 h.

### 3.2. Thermal and Thermo-Mechanical Properties of High-T_g_ Epoxy-Anhydride Vitrimers

The different cure mechanism of catalysed and non-catalysed systems together with the varying degree of homopolymerisation and residual monomers are expected to affect the thermomechanical properties of the networks. The *T*_g_ values were determined from DMTA measurements by taking the maxima of the tan δ curve and are provided in Table 2. In particular, curing of glutaric anhydride with 4-DGA yields networks with higher *T*_g_ than the counterparts cured with 3-DGOA, which is explained by the higher functionality and rigidity of the tetra-functional epoxy monomer. Independent of the epoxy monomer, the *T*_g_ of non-catalysed networks is higher than the catalysed ones. This behaviour was also found in other epoxy-anhydride networks and is related to a faster curing of the resin in the presence of a catalyst, which affects the network structure and leads to a lower cross-linking density [34,37]. With increasing concentration of the catalyst, this effect is more pronounced and a steady decrease of the *T*_g_ values is obtained [38].

**Figure 3:**
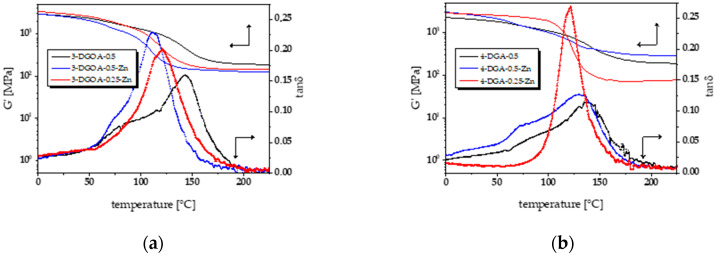
Storage modulus (*G*’) and tan δ curves of epoxy-anhydride vitrimers from (**a**) 3-DGOA and (**b**) 4-DGA.

Interestingly, catalysed 3-DGOA networks cured with *r* = 0.25 show a higher *T*_g_ than networks with *r* = 0.5. This is explained by the homopolymerisation of the epoxy monomers, which is more pronounced at a higher excess of epoxy moieties and is catalysed by the –OH groups formed during the ring opening reaction between anhydride and epoxy moieties [27]. The additionally formed ether crosslinks further shift the *T*_g_ of the networks to higher values. However, in the respective DMTA curve, only a one-step change in modulus and a single peak in tan δ is observed, which indicates a rather homogeneous network structure (Figure 3a).

This is in good agreement with previous work on epoxy-anhydride vitrimers prepared from rigid epoxy monomers [23].

In contrast, non-catalysed 3-DGOA networks prepared with *r* = 0.5 show a significantly broader peak in tan δ and a shift of the maximum tan δ to higher values. The higher heterogeneity of the networks is explained by the different cure mechanism of non-catalysed resins favouring the homopolymerisation of the epoxy monomers. Thus, non-catalysed networks are expected to have an even higher number of ether crosslinks than catalysed ones, leading to an additional transition at higher temperatures. The results suggest that the degree of homopolymerisation increases in the order of 3-DGOA-0.5-Zn < 3-DGOA-0.25-Zn < 3-DGOA-0.5.

A comparable behaviour and shift in the maximum tan δ values is also observed in the DMTA curves of non-catalysed 4-DGA networks (Figure 3b). However, an onset of a sub-glass transition is observed at around 20 °C, which indicates an incompletely cured network. 4-DGA-0.5-Zn systems follow the same trend giving rise to an incomplete homopolymerisation of the epoxy monomers. This is also corroborated by FT-IR data showing a lower number of –OH groups and a higher content of non-reacted epoxy moieties.

In contrast, 4-DGA-0.25-Zn networks are characterized by a narrow peak in the tan δ curve without an onset of a sub-glass transition region, giving rise to a highly homogeneous network structure. This behaviour is also observed in networks from 3-DGOA, as the DMTA data indicate the highest homogeneity in 3-DGOA-0.25-Zn systems. Thus, the results suggest that the network structure is comprised of well intertwined epoxy-anhydride and homopolymerised epoxy domains, since only a single and narrow peak is observed in the tan δ curve. Interestingly, the *T*_g_ of 4-DGA-0.25-Zn systems is lower than the *T*_g_ of 4-DGA-0.5-Zn networks, although a higher content of epoxy monomers is expected to yield a higher degree of homopolymerization and thus a higher content of ether linkages. This might be explained by diffusion limitations of the tetra-functional epoxy monomers, which lead to incomplete monomer conversions, as shown in the FT-IR studies, and highly heterogeneous network structures as obtained from the broad tan δ curve of 4-DGA-0.5-Zn systems. We assume that diffusion limitations of 4-DGA lead to heterogeneous networks, which contain homopolymerised epoxy domains with higher chain lengths than in 4-DGA-0.25-Zn networks. The higher chain length might facilitate the formation of separate domains of homopolymerised 4-DGA, which not only result in a broader tan δ curve but also in a stronger shift of the maximum of the tan δ curve to higher temperatures, even at a lower content of epoxy monomers.

**Figure 4:**
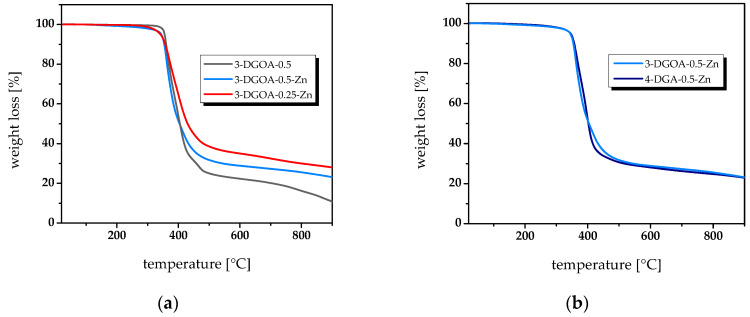
TGA curves of epoxy-anhydride vitrimers. (**a**) Influence of catalyst and stoichiometry on the thermal stability of vitrimers from 3-DGOA and glutaric acid and (**b**) influence of the type of epoxy monomer (3-DGOA vs. 4-DGA) on the thermal stability of catalysed vitrimers (epoxy and anhydride groups in stoichiometry).

Along with thermo-mechanical properties, the thermal stability of the epoxy-anhydride networks as a function of stoichiometry, catalyst and type of epoxy monomer was determined using TGA (Figure 4). By adding Zn(acac)_2_−H_2_O as catalyst, 3-DGOA-0.5 systems show a decrease in initial weight loss temperature (*T*_i_) from ~320 to 280 °C together with an increase in weight loss at 900 °C (*w*_900_) from 11% to 23%. The higher thermal stability of non-catalysed systems is related to the higher degree of homopolymerisation, as observed in DMTA experiments. Since ether groups comprise a higher thermal stability than ester moieties, the thermal properties of the related networks are improved [23]. This is also confirmed by the even higher thermal stability of catalysed networks prepared with *r* = 0.25 (3-DGOA-0.25-Zn). The further increase in both *T*_i_ (from ~280 to 300 °C) and *w*_900_ (from 23% to 28%) is mainly related to the higher number of ether crosslinks, formed at an excess of epoxy monomers in the resins formulation.

In contrast to catalyst and stoichiometry, the type of diglycidylaniline derivative used in the present study has no significant influence on the thermal stability of the epoxy-anhydride networks. Although DMTA curves indicate a higher heterogeneity in 4-DGA-anhydride networks, the weight loss curves of networks from 4-DGA and 3-DGOA are comparable and exhibit a one-stage decomposition process. As an example, Figure 4b compares the weight loss curves of catalysed networks from 4-DGA and 3-DGOA, and both networks have nearly identical *T*_i_ and *w*_900_ values.

### 3.3. Thermally Adaptable Properties and Reprocessability of High-T_g_ Epoxy-Anhydride Vitrimers

**Figure 5:**
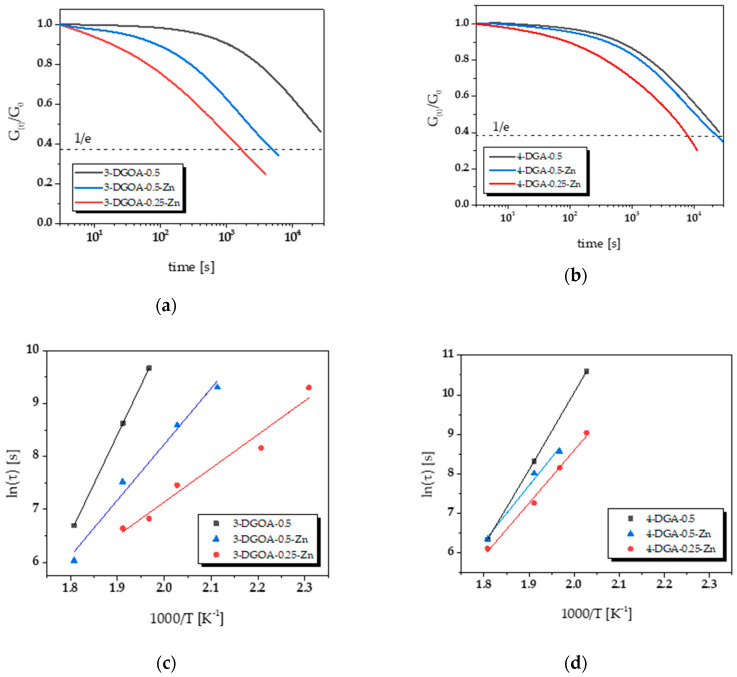
Normalised stress relaxation curves of epoxy-anhydride networks from (**a**) 3-DGOA and (**b**) 4-DGA obtained at 220 °C. Arrhenius plot of the measured relaxation times from epoxy-anhydride networks obtained with (**c**) 3-DGOA and (**d**) 4-DGA.

The thermally triggered topology rearrangements of the networks were studied using stress relaxation experiments. Since the epoxy-anhydride vitrimers under investigation comprised a *T*_g_ in the range between 113 and 140 °C and were stable at least up to 280 °C, the stress relaxation experiments were carried out between 160 and 280 °C. Consequently, thermal degradation of the networks was avoided during the rheological measurements, whilst a high mobility of the chain segments was ensured as the studies were carried out above the networks’ *T*_g_. In Figure 5a,b, the evolution of the relaxation modulus at 220 °C as a function of time is shown for 3-DGOA and 4-DGA networks, respectively. All networks comprise a distinctive stress relaxation, even networks without Zn(acac)_2_−H_2_O, albeit at a lower relaxation rate. Thus, the results clearly show that the tertiary amines of the epoxy monomers are able to catalyse the transesterification exchange reaction in the networks. As expected, the relaxation rate increases with the addition of Zn(acac)_2_−H_2_O giving rise to acceleration of the bond exchange reactions in the presence of the catalyst. In addition, networks prepared with *r* = 0.25 comprise a higher relaxation rate than the ones containing a lower excess of epoxy groups (*r* = 0.5). This is mainly attributed to the higher degree of epoxy homopolymerisation leading to a higher number of terminal –OH groups in the networks, which is well known to be advantageous for efficient stress relaxation [18]. In contrast, the limited availability of –OH groups leads to the lower relaxation rate observed in 4-DGA networks. FT-IR experiments have already showed that 4-DGA networks suffer from a lower content of –OH groups, which clearly affects the rate of bond exchange reactions at elevated temperature.

Upon determining the stress relaxation curves at different temperatures, the topology freezing temperature (*T*_v_) can be calculated from the respective Arrhenius plot [1]. Below *T*_v_, the networks behave like thermosets since the exchange reaction rate is very low and the dynamic bonds are essentially frozen. Increasing the temperature above *T*_v_, the transition from an elastic solid to a viscoelastic liquid occurs. The viscosity gradually decreases with temperature and follows the Arrhenius law. Following the Maxwell model, the relaxation times (τ*) were determined as the time needed to relax to 1/e of the initial stress. Since the τ* of vitrimers follow the Arrhenius law with the temperature, it can be expressed as *τ*_(T)_ = *τ*_0_ exp(*E*_a_/RT). *T*_v_ values are obtained by extrapolation from the Arrhenius plot [4]. The *T*_v_ values of the epoxy-anhydride networks under investigation are shown in Table 2, and the related Arrhenius plots of the relaxation times are provided in Figure 5c,d.

The activation energy (*E*_a_) for the topology freezing and the *T*_v_ values significantly differ for the networks investigated. As already observed for the stress relaxation curves at 220 °C, the bond exchange reactions are accelerated by Zn(acac)_2_−H_2_O and an increasing number of –OH groups in the network, which leads to a decrease in both *T*_v_ and *E*_a_. The high number of –OH groups might also explain the comparatively low *T*_v_ of the 3-DGOA-0.25-Zn network, which is in the range of the *T*_g_ of the network. In contrast, all other networks comprise a *T*_v_ well above the *T*_g_.

This behavior might be mainly related to the different degrees of homopolymerisation of the epoxy monomers in the networks. On the one hand, homopolymerisation leads to the formation of polymer chains with terminal –OH groups, which facilitate the transesterification and lead to lower *T*_v_ values. On the other hand, the higher number of ether bonds formed by homopolymerisation shifts the *T*_g_ of the networks to higher values. In 3-DGOA-0.25-Zn networks, this effect is particularly pronounced, leading to a *T*_v_ value which is lower than the *T*_g_. In contrast, in 4-DGA-0.25-Zn networks, the number of –OH groups is significantly lower, as shown by FT-IR data. Thus, a *T*_v_ value (188 °C) is obtained, which is higher than the *T*_g_ of the network (122 °C). Although non-catalysed networks comprise a higher degree of homopolymerisation than the catalysed counterparts, they exhibit significantly higher *T*_v_ values of 238 (4-DGA-0.5) and 241 °C (3-DGOA-0.5). The distinctive increase in *T*_v_ is mainly related to the different reactivity of organic zinc salts compared to tertiary amines in the catalysis of the transesterification reaction. Thus, even if the non-catalysed networks are characterized by the highest *T*_g_ (up to 140 °C), the values are not able to exceed the corresponding *T*_v_ data. Similar behavior of vitrimers was previously discussed by Du Prez and co-workers in their study on the material characteristics of vitrimers [2].

**Figure 6:**
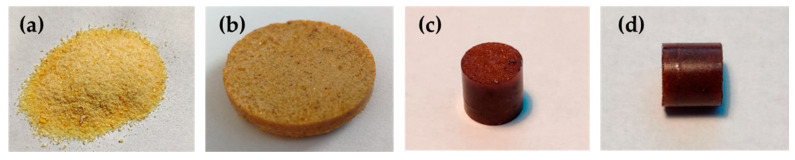
(**a**) Grinded powder from 3-DGOA-0.5-Zn networks. (**b**) Remolded test specimen obtained by applying 5 bar at 220 °C. The diameter of the disc is 25 mm. (**c**,**d**) Remolded test specimen obtained by applying 500 bar at 250 °C. The diameter of the cylinder is 7 mm.

**Figure 7:**
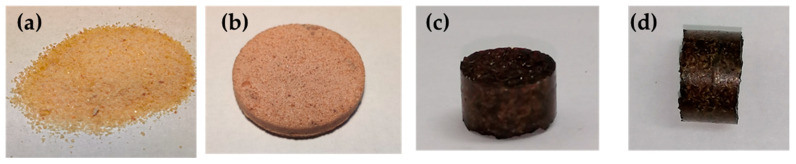
(**a**) Grinded powder from 4-DGA-0.5-Zn networks. (**b**) Remolded test specimen obtained by applying 5 bar at 220 °C. The diameter of the disc is 25 mm. (**c**,**d**) Remolded test specimen obtained by applying 500 bar at 250 °C. The diameter of the cylinder is 7 mm.

The dynamic nature of the networks was exploited to thermally reprocess the epoxy-anhydride systems. To demonstrate the recyclability of the networks, cured test plates of 3-DGOA-0.5-Zn and 4-DGA-0.5-Zn were grinded to a fine powder (Figure 6a and Figure 7a), which was remolded in a press at 5 bar and 220 °C. The grain boundaries of the resin particles were still visible in the remolded test specimen (Figure 6b and Figure 7b). However, the majority of the grain boundaries vanished (Figure 6c,d and Figure 7c,d) if the molding was carried out both at higher temperature (250 °C) and pressure (500 bar). In future work the mechanical properties and ageing of the various networks will be tested as a function of the applied recycling parameters.

## 4. Conclusions

Epoxy-anhydride vitrimers were prepared using thermal curing of tri- and tetra-functional aminoglycidyl epoxy monomers with glutaric anhydride. Cured networks were obtained at 120 °C without the addition of a catalyst, since the tertiary amine groups present in the structure of the epoxy monomers were able to accelerate the nucleophilic ring-opening reaction. Final monomer conversions decreased with rising functionality of the epoxy monomers due to diffusion limitations of the monomers at high degrees of conversion. By adding Zn(acac)_2_−H_2_O as transesterification catalyst, the curing was significantly accelerated. However, incomplete conversion of the epoxy groups was observed in networks with a high excess of epoxy monomer (*r* = 0.25). A higher content of epoxy monomer gave rise to the formation of ether linkages via homopolymerisation, which shifted the *T*_g_ and the thermal stability of the networks to higher values. The highest *T*_g_ (140 °C) was obtained with non-catalysed 4-DGA/glutaric anhydride systems, which were also able to relax stresses adequately. The results evidence that the tertiary amine groups within the structure of the epoxy monomers catalyse the transesterification exchange reaction, albeit at lower rates than networks with Zn(acac)_2_−H_2_O. Along with the zinc salt, the relaxation rate increased with rising degree of homopolymerisation, which is related to a higher number of terminal –OH groups in the networks. In particular, fast stress relaxation at a low *T*_v_ (113 °C) was obtained in catalysed 3-DGOA/glutaric anhydride networks prepared with *r* = 0.25. Furthermore, the thermally activated bond exchange reactions were exploited to reprocess the cured networks. Successful remoulding of a grinded powder was demonstrated, and in further work the influence of the reprocessing parameters on mechanical performance and ageing stability will be studied in detail. The high *T*_g_ paired with the efficient stress relaxation and ability towards reprocessing makes this type of epoxy-anhydride vitrimers interesting candidates for structural applications operating at high service temperature.

## Figures and Tables

**Table 1:** Composition of the investigated resin formulations. *r* denotes the stoichiometric ratio, which was defined as *r* = anhydride equiv/epoxy equiv.

System	3-DGOA (g)	4-DGA (g)	Anhydride (g)	Zn(acac)_2_-H_2_O (mg)	*r*
**3-DGOA-0.5-Zn**	5	-	5.7	658	0.5
**3-DGOA-0.25-Zn**	5	-	2.9	658	0.25
**3-DGOA-0.5**	5	-	5.7	-	0.5
**4-DGA-0.5-Zn**	-	5.7	5.7	658	0.5
**4-DGA-0.25-Zn**	-	5.7	2.9	658	0.25
**4-DGA-0.5**	-	5.7	5.7	-	0.5

**Table 2:** Characteristic properties of epoxy-anhydride vitrimers under investigation: *T*_g_ values derived from dynamic-mechanical thermal analysis DMTA measurements and *T*_v_ and *E*_a_ data obtained from Arrhenius plots, in which R^2^ denotes the coefficient of determination.

System	*T*_g_ (°C)	*T*_v_ (°C)	*E*_a_ (kJ/mol)	*R^2^*
**3-DGOA-0.25-Zn**	120	113	53	0.97
**3-DGOA-0.5-Zn**	112	190	87	0.95
**3-DGOA-0.5**	125	241	154	0.99
**4-DGA-0.25-Zn**	122	188	111	0.99
**4-DGA-0.5-Zn**	130	227	158	0.96
**4-DGA-0.5**	140	238	161	0.99
